# Ego States of nurses working in psychiatric clinics according to transactional analysis theory

**DOI:** 10.12669/pjms.322.9426

**Published:** 2016

**Authors:** Melike Yonder Ertem, Ayla Kececi

**Affiliations:** 1 Melike Yonder Ertem, MSc, PhD Student. Research Assistant, Department of Psychiatric Nursing, Dokuz Eylul University Nursing Faculty, Izmir, Turkey; 2 Dr. Ayla Kececi, PhD, RN. Associate Professor, Department of Nursing Education, Duzce University School of Health, Duzce, Turkey

**Keywords:** Transactional Analysis, Psychiatric Clinic, Ego States, Psychiatric Nursing, Nurse-Patient Interaction

## Abstract

**Objective:**

An effective interpersonal communication is an essential nursing skill required to help provide quality health care and meet the treatment objectives. The aim of this study was to investigate the communication between the psychiatric nurses and the patients in terms of Transactional Analysis Theory ego states.

**Methods:**

The quantitative and qualitative research methods were used. The descriptive statistics (frequency, percentage, mean, standard deviation) were used in the data analysis and Kendall’s Tau-c coefficient was used to assess the agreement among the observers.

**Results:**

Of the psychiatric nurses, 66.7% (n = 14) had served as a psychiatric nurse for 1-10 years. Among the nurses, 52.4% (n=11) had received training about communication from any institution/organization. The agreement among the opinions of the nurses, the researcher and the charge nurses about the psychiatric nurses’ ego states showed that there was a significant relationship between the researcher’s opinion of the nurses’ ego states and the charge nurses’ opinion of the nurses’ ego states in terms of Critical Parent, Nurturing Parent, Adult, Adapted Child and Natural Child ego states.

**Conclusion:**

It is suggested that training be offered in regards to raising awareness about ulterior transactions that can affect communication negatively, patient autonomy and therapeutic communication in particular, and patients requiring the use of special communication methods.

## INTRODUCTION

In the psychiatric nursing, communication skills are of vital importance, because it is possible to understand and extract information about the patient’s concerns, needs and/or problems by engaging in therapeutic interaction.^1^, ^2^ Psychiatric patients, unlike any other patient profiles, can suffer from functional disability, cognitive-perceptual changes, orientation disorders, changes in thought content and vice versa. In these cases, they can have difficulty making the right decision for themselves about the treatment and care.^3^ Therefore, attitude and behaviour of the psychiatric nurses are very important for providing quality health care services.^4^ Nurses with positive attitude relax the patients, promote their integration into the environment and thus facilitate their response to the treatment. On the contrary, negative nurse attitudes result in poor social interactions with the patients. Consecutively, patients progressively disconnect from their environment and develop augmented levels of resistance to the treatment.^5^

According to Transactional Analysis theory, an individual’s personality comprise of three ego states: Parent, Child and Adult. According to the functional analysis, the Parent ego state is divided into Critical Parent and Nurturing Parent, and the Child ego state is divided into Adapted Child and Natural Child; however, the Adult ego state remains the same.^6^-^8^ In interpersonal communications, the mutual exchange of the ego states is referred to as transactions.^7^, ^9^ Berne (1961) distinguishes three basic types of transactions: complementary, crossed and ulterior.^10^

Attitude awareness of the healthcare professionals towards patients with mental illness is critical in benefiting early and accurate diagnosis of the mental illnesses, and fulfillment of preventive treatment, care and adequate rehabilitation.^11^, ^12^ The attitude of nurses towards the mental patients sets the standards for the provided health service. In this respect, an effective interpersonal communication is not only the focus of nursing care services, but it is an essential nursing skill required to help provide quality health care and meet the treatment objectives.^4^, ^13^

The aim of this study was to investigate the communication between the psychiatric nurses and the patients in terms of Transactional Analysis Theory ego states and determine the most commonly used ego state in a nurse-patient communication.

## METHODS

### Research Design

This is a descriptive study designed as a multiple triangulation (multiple resource study comprised of qualitative and quantitative methods).

### Population and Sample

The initial population of the study consisted of 36 nurses working in the psychiatric departments of a state hospital and a university hospital. A total of 21 nurses agreed to participate in the study.

### Data Collection Process

Data were collected between February and May, 2011. In the study, the quantitative and qualitative research methods were used concurrently by administering the forms and the observations.

### Quantitative Data Collection

The Personal Information Form and the Ego State Scale were used together to collect the quantitative data. Personal Information Form was used to determine the each participant’s socio-demographic characteristics and solicit information about the intra-department communication. Then the Ego State Scale was utilized to determine the primary ego states used by the nurses in their communication with patients in the department. The Ego State Scale was also used by the charge nurses and the researcher to assess the participants.

### Qualitative Data Collection

The qualitative data in the study were collected by means of direct participant observation. All participants were assessed and observed individually. The observations were recorded on an agenda which was kept hidden by researcher. Through field observations in the corresponding departments, the researcher observed the nurses’ communication with the patients in the comfort of their natural workspace environment in the concept of Transactional Analysis theory.

### Data Analysis

The descriptive statistics (frequency, percentage, mean, standard deviation) were used in the data analysis and Kendall’s Tau-c coefficient was used to assess the agreement among the observers. The analysis carried out to determine the nurses’ ego states shows that the researcher and charge nurses are in agreement in all ego states. The nurses were coded as ‘Nu’ and patients as ‘Pa’.

## RESULTS

The results of this study are presented in two separate parts as quantitative and qualitative findings.

### Results of Personal Characteristics

Among the psychiatric nurses in the study, the mean age was 32.28 ± 4.03, 66.7% (*n* = 14) were female, 61.9% (*n* = 13) were married, 52.4% (*n* = 11) had children, 38.1% (*n* = 8) held a bachelor’s degree, and 52.4% worked in the level 1 service (acute service). Of the psychiatric nurses, 52.4% (*n* = 11) had worked as a nurse for 11-20 years, 66.7% (*n* = 14) of the participants had served as a psychiatric nurse for 1-10 years and 47.6% (*n* = 10) stated that they were content being a nurse. Among the nurses, 52.4% (*n* = 11) had received training about communication from any institution/organization.

### Results of Ego States

**Fig.1:**
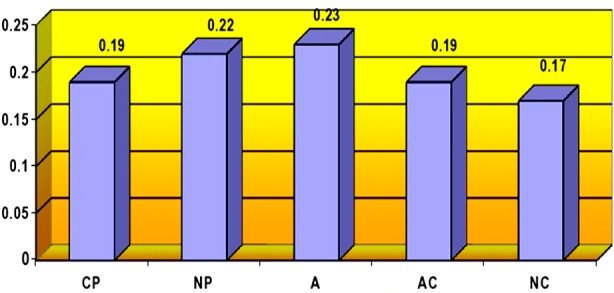
Ego states of nurses according to researcher.

**Fig.2:**
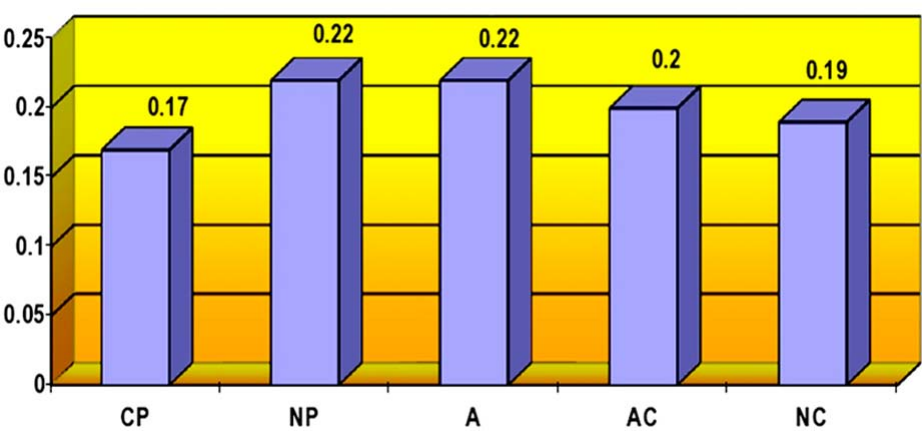
Ego States of nurses according to charge nurses.

**Fig.3:**
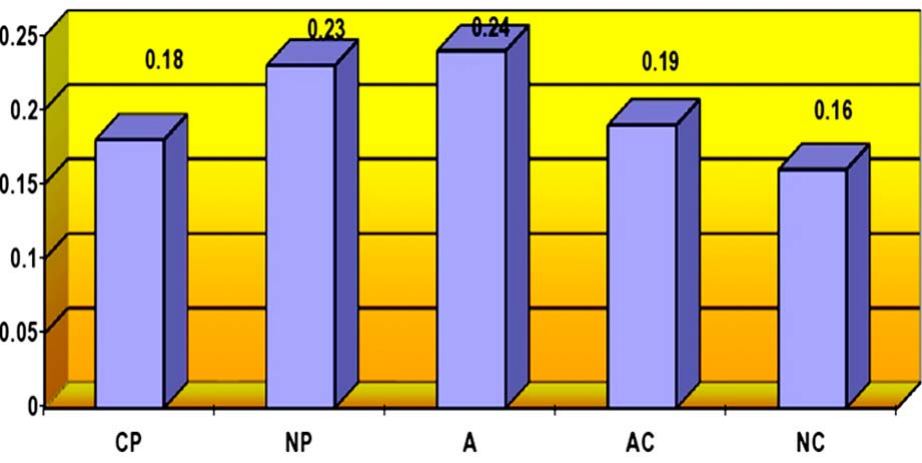
Ego states of nurses according to themselves.

Fig. 1, 2 and 3 shows the mean point of ego states of nurses according to researcher, charge nurses and psychiatric nurses themselves.

According to the researcher, the nurses primarily exhibited the Adult ego state (X±SD=0.23±0.02), whereas they exhibited the Natural Child ego state least (X±SD=0.17±0.06) (Fig.1).

The results as shown in Fig.2, the nurses primarily used the Nurturing Parent ego state (X±SD=0.22±0.05) and the Adult ego state (X±SD=0.22±0.03), whereas they used the Critical Parent ego state least (X±SD=0.17±0.07) according to charge nurses.

**Table-I:** Agreement among the opinions of the nurses, the researcher and the charge nurses about the psychiatric nurses’ ego states.

		Nurses	Charge Nurses	Researcher

		Researcher	Nurses	Charge Nurses
CP	Kendall’s Tau-c	0.343	0.219	0.400
	P	0.018	0.107	0.007
NP	Kendall’s Tau-c	0.448	0.333	0.448
	P	0.001	0.004	0.002
A	Kendall’s Tau-c	-0.010	0.124	0.524
	P	0.952	0.454	0.000
AC	Kendall’s Tau-c	0.467	0.495	0.533
	P	0.001	0.000	0.000
NC	Kendall’s Tau-c	0.162	0.381	0.629
	P	0.275	0.028	0.000

CP: Critical ParentNP: Nurturing ParentA: AdultAC: Adapted ChildNC: Natural Child

According to the psychiatric nurses themselves, they primarily exhibited the Adult ego state (X±SD=0.24±0.02), whereas they exhibited the Natural Child ego state least (X±SD=0.16±0.04) (Fig.3).

The agreement among the opinions of the nurses, the researcher and the charge nurses about the psychiatric nurses’ ego states showed that there was a significant relationship between the researcher’s opinion of the nurses’ ego states and the charge nurses’ opinion of the nurses’ ego states in terms of Critical Parent (Kendall’s Tau-c = 0.400; *p*< 0.05), Nurturing Parent (Kendall’s Tau-c = 0.448; *p*< 0.05), Adult (Kendall’s Tau-c = 0.524; *p*< 0.05), Adapted Child (Kendall’s Tau-c = 0.533; *p*< 0.05) and Natural Child (Kendall’s Tau-c = 0.629; *p*< 0.05) ego states. This result indicates the similarity between the assessments made by the researcher and the charge nurses for all of the ego states.

### Results of Qualitative Data

The qualitative data in this study were obtained using the direct observation method. The following are samples for each type of transaction.

**Table:** 

*Complementary Transactions*
*Pa_1_*: Are we allowed to smoke?
*Nu_1_*: Yes, you are. Smoking hours are three times a day and the hours are …

The nurse gave information about the service with explanatory and clear statements and made direct eye contact with the patient during the interaction. The nurse’s tone of voice was calm, safe and direct. In addition, during this interaction, the nurse’s posture was directed at the patient and the nurse applied the procedure required for patient admission (field notes).

**Table:** 

*Ulterior Transactions*
*Pa_30_*: When can I leave? I’ve got things to do outside (the patient gave the impression “Save me from here”). *Nu_7_*: What will you do outside? (The nurse asked this in a direct, sharp, calm manner and with a wry smile as if trying to say, “What on earth could someone like you do outside?”). *Pa_30_*: I need to get a job, I’m going to earn money, and my mother is alone. *Nu_7_*: All right, what else do you have to do outside?

Listening to the patient, the nurse asked the patient some questions, but with a smile. The patient paused thoughtfully while talking. The nurse listened to what the patient said but seemed to be looking down on the patient because of the smile on the nurse’s face (*field notes*).

**Table:** 

*Crossed Transactions*
*Pa_34_*: When will my doctor be here? *Nu_4_*: Stop asking! I do not know when your doctor will come (closed the window saying that).

The posture of Nu_4_ was directed at the patient, but the nurse was busy on a computer with patients’ processes and did not look at the patient’s face. The patient directly asked the nurse a question with a calm tone of voice, but the nurse seemed to have a careless attitude towards the patient (*field notes*).

## DISCUSSION

The psychiatric nurses primarily used the Adult and Nurturing Parent ego states according to the researcher, the charge nurses and the psychiatric nurses. In a study by Akbag and Deniz (2003), the ego states used in communication processes between faculties and university students were determined as Adult and Nurturing Parent as well.^14^ The frequent use of the Adult and Nurturing Parent ego states is an indicator for exhibiting effective approaches during communication process. The Adult ego state is associated with the ability to generate ideas, use problem-solving skills, adhere to the principle of equality and justice, share power and authority, build confidence, prioritize personal development, promote learning (active listening, learning from errors), integrity, collaboration, critical/creative thinking, and improve the quality and efficiency of life (at both the individual and societal level).

The Nurturing Parent ego state, on the other hand, is associated with rewarding achievements, presenting opportunity, leading improvement of others, courage, honesty and showing sincerity.^15^ Bostanci, Asti (2000) and Pestonjee, Sharma and Patel (2005) found that nurses mostly used problem solving as a component of the Adult ego state, providing support as a component of the Nurturing Parent ego state, and being flexible as a component of the Adapted Child.^1^, ^16^ However, this result is not supported by Isik’s (2010) finding that nurses working in psychiatric departments and doctors working in non-psychiatric departments often exhibited negative opinions and attitudes towards individuals with mental disorders as a component of the Critical Parent ego state.^12^ Also, Moran (2008) found that the average score of authority, which is a component of the Critical Parent ego state, of nurses working in psychiatric departments was higher than that of nurses working in non-psychiatric departments and this difference was significant.^4^

Despite the different results concerning nurses’ communication with patients, the Critical Parent ego state is reported to be used because psychiatric patients are more dependent and it is more convenient for them to leave their problems to health personnel. Nurses may sometimes act on instinct to control dependent patients who need their care.^17^ Patients are expected to obey what they are told like a child, follow orders and behave themselves.^13^ Those patients who are demanding or ask questions, express their wishes, refuse some processes and so on are called problem patients by health personnel. These so-called problem patients may enter a kind of regression process in order to cope with their fears and concerns and feel safe and then they may primarily exhibit the Child Ego state.^13^ This case may occur in schizophrenia, depression and anxiety disorders.^18^ The relevant literature often points out that nurses should support patients’ independence as they make progress.^17^, ^19^ Transactional Analysis theory holds that even though each ego state’s contribution is needed in the communication process, it is recommended that reactions be under the control of the Adult ego state.^14^ Such an individual possesses some characteristics like sincere interest and judgments about others as a part of the Nurturing Parent ego state, problem-solving skills as a part of the Adult ego state and the creativity and curiosity exhibited by a healthy Child.^14^, ^20^ In this sense, the use of Adult and Nurturing Parent ego states rather than the Critical Parent ego state by the nurses giving care to psychiatric patients and the agreement among the participants, and other observers about their assessments of the nurses’ ego states could be considered as favourable.

**Table:** 

*Pa_12_*: They tell me bad things but I’m trying not to listen to them (in a nervous and frightened manner). *Nu_18_*: Yes. You’re not going to listen to them and you are going to be strong. If they disturb you, you’ll inform your doctor (in a bossy manner pointing index finger to the patient). *Pa_12_*: All right. I’m going to try to be strong and tell my doctor. *Nu_18_*: Yes, that’s exactly what you should do.

The nurse exhibited a caring-protective attitude and an encouraging and paternalistic approach towards the patient with auditory hallucinations. In the meantime, the nurse did not comment on “what the voices that the patient heard told the patient, how often the patient heard these voices, what the patient did to cope with these voices, or what else could be done”.

There was a significant relationship between the ego states of the nurses according to the nurses, researcher and the charge nurses in terms of Critical Parent, Nurturing Parent, Adult, Adapted Child and Natural Child. The assessments made by the researcher and the charge nurses are similar in all of the ego states. The results of this study could be taken to suggest that in this kind of observation study, a minimum of three hours and a maximum of nine hours could be sufficient as a duration allocated for assessing one person.

In light of the qualitative data obtained by using the direct observation method, the psychiatric nurses’ interactions with male or female patients diagnosed with depression, alcohol addiction, schizophrenia, anxiety, bipolar affective disorder, atypical psychosis and depression, mental retardation, or psychosis were analysed. It was observed that the nurses’ interactions with patients were affected by some factors such as patients’ cognitive state, their tendency towards aggression, nurses being alone or with few people at the desk, nurses having heavy workloads and so on.

The sample above shows that the communications varied depending on patients’ cognitive state and the Critical Parent ego state was used in the case above. In the sample, a patient told the nurse about the auditory hallucinations and asked for help on this issue and the nurse, by using the Critical Parent ego state, told the patient to adopt a strong and persistent attitude and gave the patient direct instructions on what to do. In the meantime, the nurse did not comment on “what the voices that the patient heard told the patient, how often the patient heard these voices, what the patient did to cope with these voices, or what else could be done”. It was observed that the principles of the therapeutic approach were not used in the case mentioned above.

### Clinical implications

On the perspective of clinical implications training be offered in regards to raising awareness about ulterior transactions that can affect communication negatively, patient autonomy and therapeutic communication in particular, and patients requiring the use of special communication methods. Awareness about using ego states and transactions during the interaction will provide an effective and efficient communication between patient and nurse.
